# Type IV Secretion-Dependent Activation of Host MAP Kinases Induces an Increased Proinflammatory Cytokine Response to *Legionella pneumophila*

**DOI:** 10.1371/journal.ppat.1000220

**Published:** 2008-11-28

**Authors:** Sunny Shin, Christopher L. Case, Kristina A. Archer, Catarina V. Nogueira, Koichi S. Kobayashi, Richard A. Flavell, Craig R. Roy, Dario S. Zamboni

**Affiliations:** 1 Section of Microbial Pathogenesis, Yale University School of Medicine, New Haven, Connecticut, United States of America; 2 Department of Immunobiology, Yale University School of Medicine, New Haven, Connecticut, United States of America; 3 Instituto de Ciencias Biomedicas Dr. Abel Salazar, Universidade do Porto, Porto, Portugal; 4 Section of Cancer Immunology and AIDS, Dana-Farber Cancer Institute and Department of Pathology, Harvard Medical School, Boston, Massachusetts, United States of America; 5 Howard Hughes Medical Institute, Yale University School of Medicine, New Haven, Connecticut, United States of America; Tufts University School of Medicine, United States of America

## Abstract

The immune system must discriminate between pathogenic and nonpathogenic microbes in order to initiate an appropriate response. Toll-like receptors (TLRs) detect microbial components common to both pathogenic and nonpathogenic bacteria, whereas Nod-like receptors (NLRs) sense microbial components introduced into the host cytosol by the specialized secretion systems or pore-forming toxins of bacterial pathogens. The host signaling pathways that respond to bacterial secretion systems remain poorly understood. Infection with the pathogen *Legionella pneumophila*, which utilizes a type IV secretion system (T4SS), induced an increased proinflammatory cytokine response compared to avirulent bacteria in which the T4SS was inactivated. This enhanced response involved NF-κB activation by TLR signaling as well as Nod1 and Nod2 detection of type IV secretion. Furthermore, a TLR- and RIP2-independent pathway leading to p38 and SAPK/JNK MAPK activation was found to play an equally important role in the host response to virulent *L. pneumophila*. Activation of this MAPK pathway was T4SS-dependent and coordinated with TLR signaling to mount a robust proinflammatory cytokine response to virulent *L. pneumophila*. These findings define a previously uncharacterized host response to bacterial type IV secretion that activates MAPK signaling and demonstrate that coincident detection of multiple bacterial components enables immune discrimination between virulent and avirulent bacteria.

## Author Summary

The host immune system senses bacterial infection by recognizing conserved bacterial components. The host can differentiate between virulent and avirulent bacteria by detecting the activity of bacterial secretion systems that inject effector proteins into cells. How the host responds to such bacterial secretion systems is not fully understood. Using the bacterial pathogen *Legionella pneumophila* and isogenic mutants that differ in defined virulence properties, a robust immune response to *L. pneumophila* and its type IV secretion system was identified. Using macrophages lacking various aspects of the innate immune system, it was found that this host response is comprised of signaling by extracellular and intracellular immune receptors, as well as host signaling triggered by the type IV secretion system. Through genomic and biochemical analyses of *L. pneumophila*–infected macrophages, type IV secretion was found to activate additional host signaling pathways distinct from known immune pathways. Our data indicate that coincident detection of multiple bacterial components is required for a robust immune response to bacterial infection and highlights a key host pathway triggered by bacterial type IV secretion that contributes to this immune response.

## Introduction

Innate immunity against bacterial pathogens is initiated by germline-encoded pattern recognition receptors (PRRs) that detect pathogen-associated molecular patterns (PAMPs) [1]. Toll-like receptors (TLRs) distinguish self from microbial non-self, but cannot distinguish pathogenic from nonpathogenic microbes. Many bacterial pathogens utilize virulence mechanisms such as active invasion into host cells, the avoidance of endolysosomal destruction, and the translocation of virulence factors into host cells through specialized secretion machinery in order to modulate host cell signaling and avoid, manipulate, or silence the immune response [2]. However, our knowledge of how innate immune cells detect the signatures of bacterial virulence and initiate appropriate immune responses remains incomplete. Recent studies show that macrophages and DCs utilize cytosolic PRRs, such as the Nod-like receptor (NLR) family, to detect bacterial products introduced into the host cytosol by bacterial secretion systems or virulent bacteria that escape to the host cytosol following host cell entry [3]–[5]. The NLRs Nod1 and Nod2 sense cytosolic peptidoglycan (PG) [6]–[10] and trigger NF-κB and MAPK signaling by a pathway that involves the signaling adaptors RIP2 [11],[12] and Card9 [13]. Additionally, several NLRs participate in inflammasome formation, leading to caspase-1 activation, processing and secretion of the cytokines IL-1β and IL-18, cell death, and cell-autonomous restriction of bacterial infection [14],[15]. For example, the NLR Ipaf responds to cytosolic bacterial flagellin [16],[17] and the NLR Naip5/Birc1e responds to bacterial type IV secretion [18], possibly by detection of cytosolic flagellin as well [19]–[21]. Furthermore, bacteria that reside in the host cytosol or vacuolar bacteria that translocate bacterial products into the cytosol activate a TLR-independent, IRF3-dependent IFNβ response [22]–[27] by a currently unknown mechanism that may involve host sensing of bacterial nucleic acids [25],[27].

In this study, *Legionella pneumophilla* was used as a model organism to dissect host responses to bacterial type IV secretion systems. *L. pneumophila* is the etiological agent of the severe pneumonia Legionnaires' disease [28]. Upon host cell entry, virulent *L. pneumophila* modulates transport of the vacuole in which it resides to prevent fusion with early and late endocytic organelles. It then recruits vesicles exiting the ER and fusion of these vesicles remodels the vacuole into an ER-derived compartment that supports bacterial replication [29]. The ability to evade endocytic maturation and create an ER-derived vacuole requires a bacterial protein secretion system encoded by the *dot* and *icm* genes [30]–[33]. The Dot/Icm type IV secretion system (T4SS) delivers effector proteins that modulate eukaryotic cellular functions into the host cytosol [34]. *L. pneumophila* mutants with a defective T4SS fail to remodel the vacuole in which they reside and undergo rapid endocytic maturation [31],[35]. Thus, comparison of wild-type (WT) *L. pneumophila* and mutant strains provides a useful model system to dissect host responses to bacteria that differ in defined virulence properties.

Innate immunity is essential for restricting *L. pneumophila* infection at the cellular and organismal level. TLRs are required for control of *L. pneumophila* infection *in vivo*, as mice lacking TLR2 are more susceptible to infection, and *Myd88* ^−/−^ mice have a profound defect in controlling *L. pneumophila* infection [36],[37]. The cytosolic NLRs Naip5/Birc1e and Ipaf activate caspase 1, leading to the processing and secretion of IL-1β and IL-18 and the cell-autonomous restriction of *L. pneumophila* replication by a mechanism requiring detection of T4S [18] and flagellin [19]–[21],[38]. Additionally, the *L. pneumophila* T4SS induces IRF-3-dependent IFNβ production by an unknown mechanism possibly involving host detection of translocated bacterial DNA [25],[26]. Interestingly, previous data indicate there is a robust multi-cytokine response to virulent versus avirulent *L. pneumophila* [39]–[41]. This cytokine response requires a functional *L. pneumophila* T4SS [39]–[41]. The basis of this response is unknown.

Here, we compared host responses to virulent *L. pneumophila* and avirulent *L. pneumophila* that are deficient in the Dot/Icm T4SS. We define a previously uncharacterized TLR- and Nod1/Nod2-independent response to *L. pneumophila* type IV secretion that activates MAP kinases and is important for a robust proinflammatory cytokine response.

## Results

### The cytokine response to *L. pneumophila* is comprised of TLR-dependent and T4SS-dependent, TLR-independent responses

**Figure 1:**
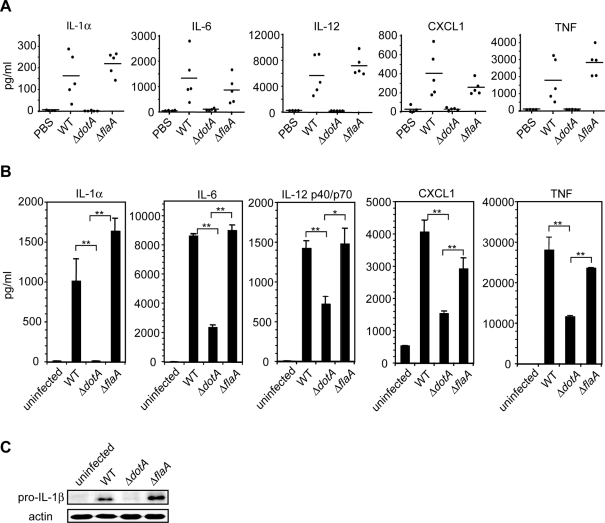
Increased cytokine production in response to *L. pneumophila* containing a functional T4SS compared to *dotA* mutants. (A) ELISA measurements of cytokine levels in the BALFs of WT mice 24 hours following intranasal infection with PBS vehicle control or 5×10^6^ CFUs of WT, Δ*flaA*, or Δ*dotA L. pneumophila* on the *thyA* background. Each point represents an individual mouse. Lines indicate the mean cytokine levels for each group of mice. (B) ELISA measurement of cytokine production in WT bone marrow-derived macrophages infected with WT, Δ*dotA*, or Δ*flaA L. pneumophila* on the *thyA* background at an MOI = 5 for 24 hours. Data represent the mean±standard error of the mean (SEM) of the assay performed in triplicate and are representative of at least three independent experiments. *P*-values derived from two-tailed student's T test. * represents *p*<0.05. ** represents *p*<0.01. (C) Immunoblot analysis of pro-IL-1β production in WT bone marrow-derived macrophages infected with WT, Δ*dotA*, or Δ*flaA L. pneumophila* on the *thyA* background at an MOI = 5 for 24 hours. Blots were reprobed for analysis of total actin (loading control). Data are representative of at least two independent experiments.

Previous data indicated that virulent *L. pneumophila* expressing a functional T4SS induce a more robust cytokine response than avirulent mutants expressing a defective T4SS [39]–[41], but the basis for this response was unclear. Following intranasal infection, we determined that WT *L. pneumophila*, but not Δ*dotA* mutant *L. pneumophila* defective in the T4SS, induced robust production of the cytokines IL-1α, IL-6, IL-12, CXCL1, and TNF (Figure 1A). This difference was independent of bacterial replication, as the bacteria used in these experiments were *thyA* mutants and fail to replicate due to their thymidine auxotrophy [31],[42]. Because C57Bl/6 mice encode a functional Naip5 allele that limits *L. pneumophila* replication [43],[44], we examined cytokine responses to flagellin-deficient *L. pneumophila*, which fail to activate this Naip5-mediated pathway [19]–[21],[38]. Infection with the Δ*flaA* strain induced a robust cytokine response equivalent to infection with WT *L. pneumophila*, indicating that this response is independent of flagellin-dependent inflammasome activation (Figure 1A).

Bone marrow-derived macrophages also produced significantly more IL-1α, pro-IL-1β, IL-6, IL-12, CXCL1, and TNF in response to WT *L. pneumophila* compared to the Δ*dotA* mutant independently of bacterial replication and cytosolic detection of flagellin (Figure 1B and 1C). Similar results were obtained in A/J macrophages (data not shown) homozygous for an allele of Naip5 that is defective in responding to *L. pneumophila* infection [43]–[45], indicating this cytokine response is Naip5-independent.

**Figure 2:**
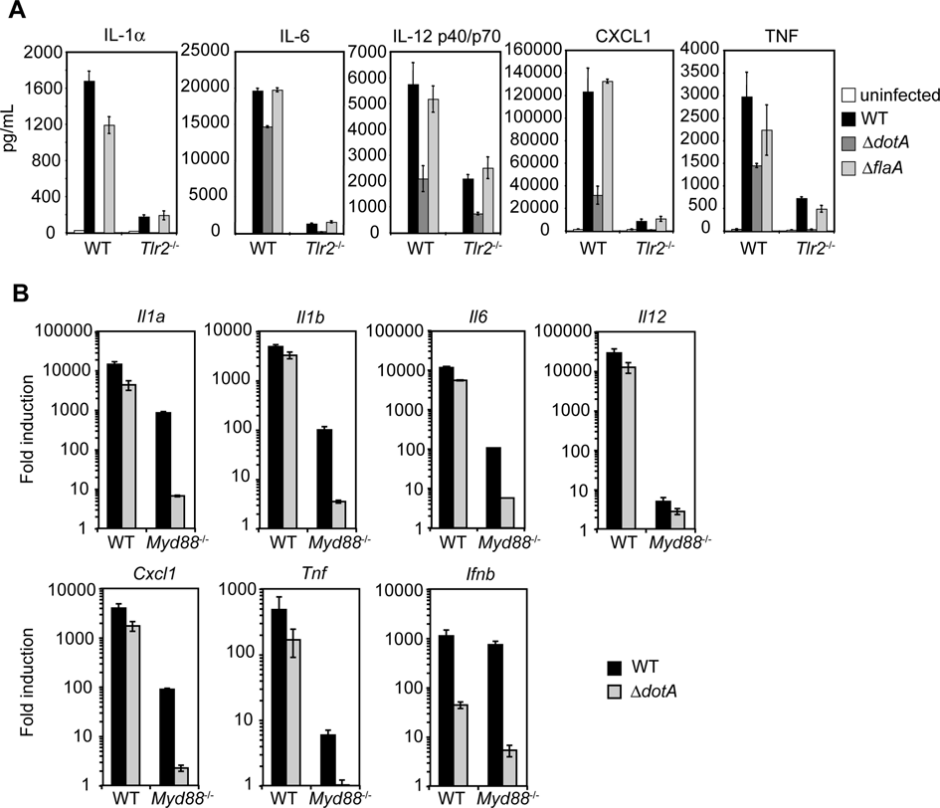
TLR-dependent signaling synergizes with TLR-independent, T4SS-dependent signaling to induce an increased cytokine response. (A) ELISA measurements of IL-1α, IL-6, IL-12 p40/p70, CXCL1, and TNF production in WT and *Tlr2* ^−/−^ bone marrow-derived macrophages infected with WT, Δ*dotA*, or Δ*flaA L. pneumophila* on the *thyA* background at an MOI = 5 for 24 hours. Data are represented as the mean±SEM of the assay performed in triplicate and are representative of at least two independent experiments. (B) Quantitative RT-PCR analysis of WT or *Myd88* ^−/−^ macrophages infected with WT, Δ*dotA*, or Δ*flaA L. pneumophila* at an MOI = 25 for four hours. Data represent the mean fold induction±SEM relative to uninfected macrophages of the assay performed in triplicate and are representative of at least two independent experiments.

Previous observations that *L. pneumophila*-infected *Tlr2^−/−^* and *Myd88* ^−/−^ macrophages produce severely diminished levels of cytokines [36] indicated that TLR signaling is required for cytokine production in response to *L. pneumophila*. *L. pneumophila*-infected *Tlr2* ^−/−^ macrophages displayed severely diminished cytokine production (Figure 2A). However, *Tlr2* ^−/−^ macrophages infected with WT or Δ*flaA L. pneumophila* still produced cytokine levels that were higher compared to macrophages infected with the Δ*dotA* mutant (Figure 2A). Cytokine production was undetectable in infected *Myd88^−/−^* macrophages (data not shown and [29]). This demonstrates that TLR signaling synergizes with T4SS-dependent host signaling to enhance cytokine production.

We then examined cytokine mRNA transcription in *L. pneumophila*-infected WT and *Myd88* ^−/−^ macrophages. Cytokine mRNA levels were increased in WT macrophages infected with WT *L. pneumophila* compared to those infected with the Δ*dotA* mutant (Figure 2B). In *Myd88* ^−/−^ macrophages, although transcription was diminished, there was still significantly increased transcription of *Il1a*, *Il1b*, *Il12*, *Cxcl1*, and *Tnf* in response to WT *L. pneumophila*, but very little transcription in response to the Δ*dotA* mutant (Figure 2B). As previously reported [25], there was also robust MyD88-independent, T4SS-dependent *Il6* and *Ifnb* transcription (Figure 2B). These data show that the cytokine response to the Δ*dotA* mutant is entirely MyD88-dependent, whereas the response to WT *L. pneumophila* is comprised of both MyD88-dependent and MyD88-independent responses. Similar results were found in macrophages lacking MyD88 as well as Trif, a signaling adaptor for TLR4 and TLR3 that leads to IRF3 activation (data not shown). Thus, maximal cytokine responses to *L. pneumophila* require both TLR detection of *L. pneumophila* and TLR-independent responses to T4S.

### RIP2-dependent and -independent responses to *L. pneumophila* type IV secretion

**Figure 3:**
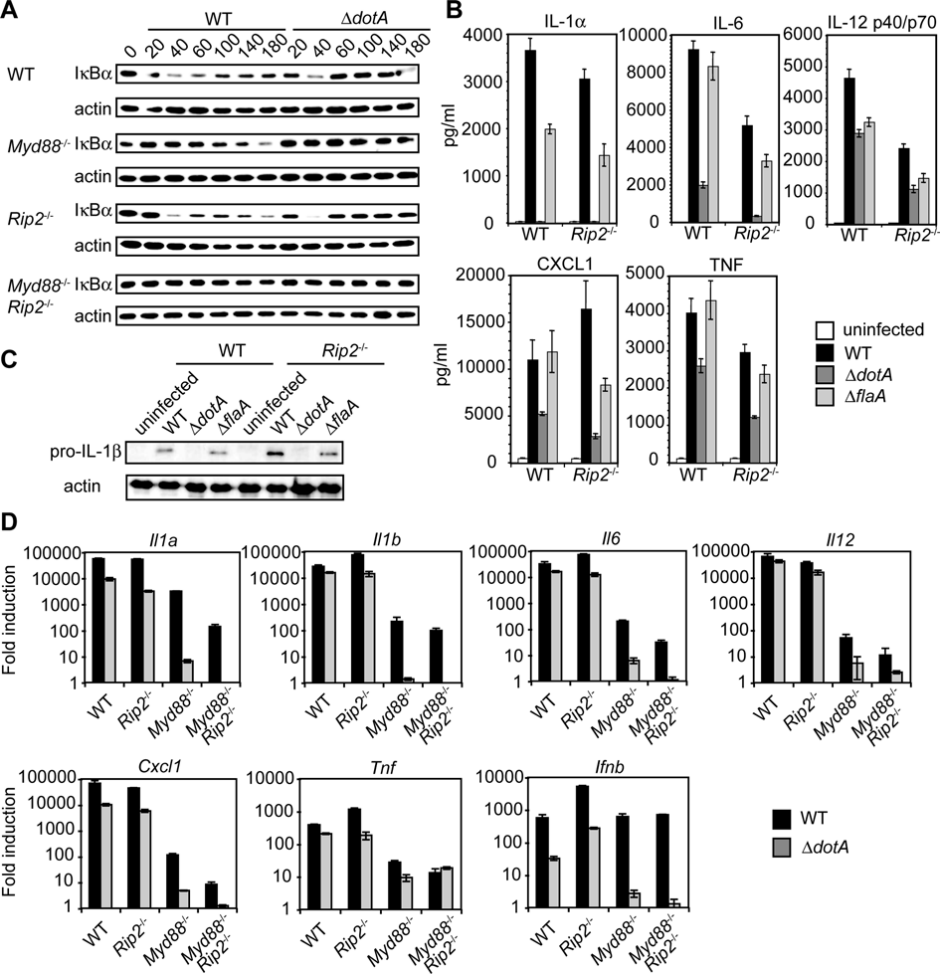
RIP2-dependent NF-κB signaling in response to *L. pneumophila* type IV secretion is not required for T4SS-dependent cytokine production. (A) Immunoblot analysis of IκB degradation in WT, *Myd88* ^−/−^, *Rip2* ^−/−^, or *Myd88* ^−/−^ *Rip2* ^−/−^ macrophages infected with WT or Δ*dotA L. pneumophila* at an MOI = 50. Blots were reprobed for analysis of total actin (loading control). Data are representative of at least three independent experiments. (B) ELISA measurements of cytokine production in WT and *Rip2* ^−/−^ macrophages infected with WT, Δ*dotA*, or Δ*flaA L. pneumophila* on the *thyA* background at an MOI = 5 for 24 hours. Data represent the mean±SEM of the assay performed in triplicate and are representative of at least two independent experiments. (C) Immunoblot analysis of pro-IL-1β production in WT and *Rip2* ^−/−^ bone marrow-derived macrophages infected with WT, Δ*dotA*, or Δ*flaA L. pneumophila* on the *thyA* background at an MOI = 5 for 24 hours. (D) Quantitative RT-PCR analysis of WT, *Rip2^−/−^*, *Myd88* ^−/−^, or *Myd88^−/−^Rip2^−/−^* macrophages infected with WT, Δ*dotA*, or Δ*flaA L. pneumophila* at an MOI = 25 for four hours. Data are represented as the mean fold induction±SEM relative to uninfected macrophages of the assay performed in triplicate and are representative of at least two independent experiments.

The NLRs Nod1 and Nod2 are cytosolic sensors of peptidoglycan [5],[46],[47]. Nod1 can detect peptidoglycan delivered by the *Helicobacter pylori* T4SS into the host cytosol [48]. Additionally, simultaneous stimulation of TLRs and NLRs induces synergistic cytokine production [10],[49],[50]. The signaling adaptors MyD88 and RIP2 control NF-κB activation downstream of most TLRs and Nod1 and 2, respectively [11],[12],[51]. Thus, we analyzed MyD88- and RIP2-dependent NF-κB activation in response to *L. pneumophila*. WT and *Rip2* ^−/−^ macrophages infected with WT or Δ*dotA L. pneumophila* displayed robust NF-κB activation, as determined by IκB degradation (Figure 3A) and NF-κB translocation into the nucleus (data not shown). In agreement with a previous study [52], in *Myd88* ^−/−^ macrophages, WT *L. pneumophila* were able to induce IκB degradation (Figure 3A and Figure S1) and NF-κB nuclear translocation (data not shown), whereas the Δ*dotA* bacteria were not. In contrast, in *Myd88* ^−/−^ *Rip2* ^−/−^ macrophages, IκB degradation (Figure 3A and Figure S1) or NF-κB nuclear translocation (data not shown) were undetectable in either WT or Δ*dotA L. pneumophila*-infected cells. Thus, there are primarily two modes of NF-κB activation in response to *L. pneumophila*, which include MyD88-dependent, T4SS-independent detection of bacterial surface structures and RIP2-dependent, T4SS-dependent detection of bacterial factors translocated into the cytosol.

We then examined whether RIP2 contributes to the T4SS-dependent cytokine response. There was a slight decrease in cytokine production by *L. pneumophila*-infected *Rip2* ^−/−^ macrophages compared to WT macrophages (Figure 3B). However, *Rip2* ^−/−^ macrophages still induced significantly increased cytokine production in response to WT *L. pneumophila* compared to the Δ*dotA* mutant (Figure 3B and 3C). *Nod1* ^−/−^ and *Nod2* ^−/−^ single knockout macrophages, and *Nod1* ^−/−^ *Nod2* ^−/−^ double knockout macrophages (Figure S2) responded similarly, indicating the presense of other cytosolic responses to T4S.

We then compared cytokine mRNA transcription in WT, *Rip2* ^−/−^, *Myd88* ^−/−^ and *Myd88* ^−/−^ *Rip2* ^−/−^ macrophages infected with WT or Δ*dotA L. pneumophila*. There was a T4SS-dependent increase in cytokine transcription in WT and *Rip2* ^−/−^ macrophages (Figure 3D). In the absence of MyD88, mRNA transcription was decreased one to three logs, but there was still significant T4SS-dependent transcriptional induction of *Il1a*, *Il1b*, *Il6*, *Il12*, *Cxcl1* (*Kc*), and *Il6* (Figure 3D). Transcription of all six genes was reduced in *Myd88* ^−/−^ *Rip2* ^−/−^ macrophages compared to *Myd88^−/−^* macrophages, indicating that RIP2 also contributes to T4SS-dependent gene transcription (Figure 3D). However, there was still significant MyD88- and RIP2-independent, T4SS-dependent gene transcription, with highly robust transcription observed for *Il1a* and *Il1b* (Figure 3D). As reported previously [25], we also observed robust MyD88- and RIP2-independent, Dot/Icm-dependent *Il6* and *Ifnb* induction. Additionally, the Δ*flaA* mutant induced cytokine transcription to the same extent as WT bacteria in *Myd88^−/−^Rip2^−/−^* macrophages (data not shown). Thus, there is an important signaling pathway that responds to the T4SS that is activated independently of MyD88, RIP2, and the inflammasome.

### A MyD88- and RIP2-independent gene expression program is induced in response to the *L. pneumophila* T4SS

**Figure 4:**
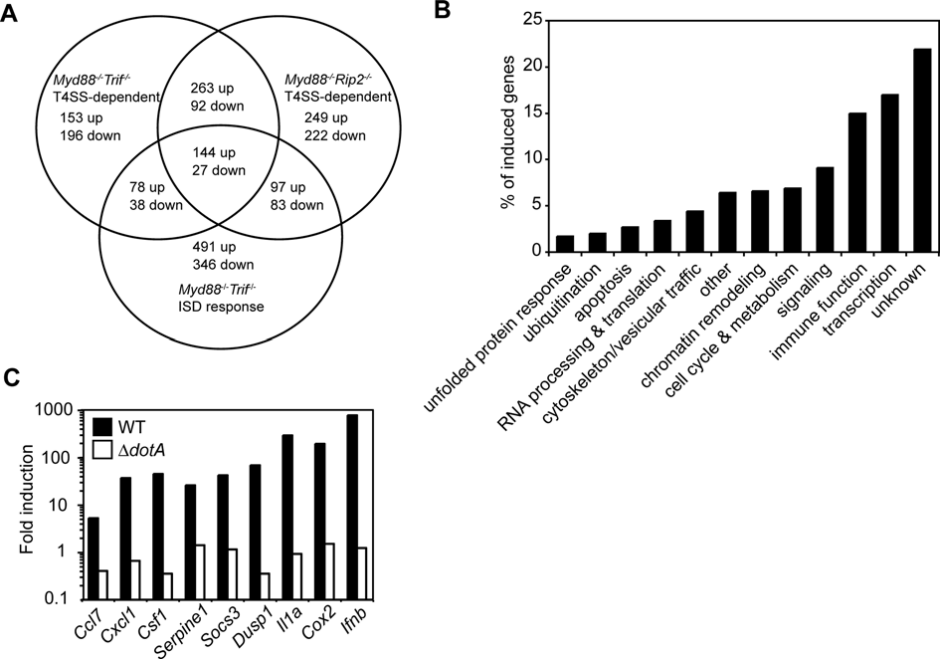
*L. pneumophila* type IV secretion induces a MyD88- and RIP2-independent transcriptional response. (A) Venn diagram showing genes transcriptionally induced two-fold or more in response to the T4SS in *Myd88^−/−^Trif^−/−^* or *Myd88^−/−^Rip2^−/−^* macrophages infected for four hours with WT *L. pneumophila* compared to those infected with the Δ*dotA* mutant on the *thyA* background at an MOI = 25 versus genes transcriptionally induced two-fold or more at four hours following ISD transfection of *Myd88^−/−^Trif^−/−^* macrophages [25]. (B) Graph representing the percentage of genes transcriptionally induced two-fold or more upon infection with WT *L. pneumophila* compared to the Δ*dotA* mutant on the *thyA* background at an MOI = 25 for four hours in both *Myd88* ^−/−^ *Trif* ^−/−^ and *Myd88* ^−/−^ *Rip2* ^−/−^ macrophages and that belong to various functional classes. (C) Quantitative RT-PCR analysis of *Myd88* ^−/−^ *Rip2* ^−/−^ macrophages infected with WT or Δ*dotA* mutant *L. pneumophila* at an MOI = 25 for four hours. Data are representative of at least two independent experiments.

To screen for additional MyD88-independent, RIP2-independent signaling pathways that respond to the T4SS, we analyzed the transcriptional responses of *Myd88* ^−/−^ *Trif* ^−/−^ or *Myd88* ^−/−^ *Rip2* ^−/−^ macrophages infected with either WT or Δ*dotA L. pneumophila*. These microarrays revealed genes whose expression were increased or decreased two-fold or more in response to WT *L. pneumophila* versus the Δ*dotA* mutant (Figure 4A and Tables S2, S3, and S4). We then focused on differentially expressed genes common to *Myd88* ^−/−^ *Trif* ^−/−^ and *Myd88* ^−/−^ *Rip2* ^−/−^ macrophages (Figure 4A and Table S2), as they were likely to represent the product of TLR- and Rip2-independent signaling induced by the *L. pneumophila* T4SS. These genes are associated with a broad spectrum of cellular functions, including immune signaling (Figure 4B). Because the *L. pneumophila* T4SS stimulates *Ifnb* transcription [25],[26] by a proposed mechanism involving T4SS-mediated translocation of bacterial nucleic acids into the host cytosol [25], we compared our analysis with the microarray analysis of the interferon stimulatory DNA (ISD) response. Although there is a subset of overlapping genes (Figure 4A and Table S5), many of the differentially regulated genes are unique to the T4SS response (Table S6).

We confirmed the MyD88- and RIP2-independent, T4SS-dependent transcription of several immune-related genes identified in the microarray analysis (Figure 4C). Transcription of these genes was independent of cytosolic detection of flagellin and Trif signaling (data not shown). Collectively, the data so far demonstrate that multiple transcriptional programs are turned on by macrophages in response to *L. pneumophila* infection: 1) T4SS-independent, MyD88-dependent gene expression; 2) T4SS-dependent, MyD88-independent, RIP2-dependent gene expression; and 3) T4SS-dependent, MyD88-independent, RIP2-independent gene expression.

### MyD88- and RIP2-independent p38 and SAPK/JNK MAPK signaling in response to *L. pneumophila* type IV secretion

**Figure 5:**
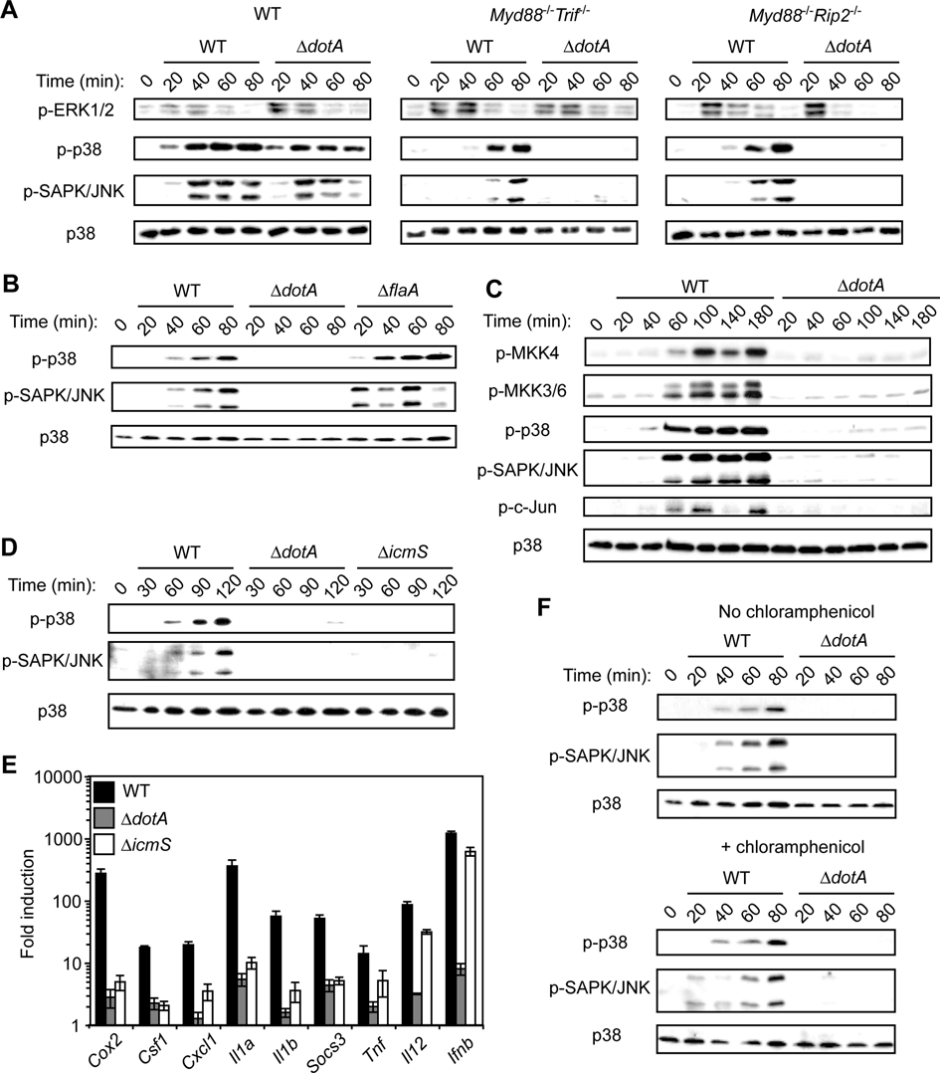
*L. pneumophila* type IV secretion induces p38 and SAPK/JNK MAPK activation independently of TLR, Nod1, and Nod2 signaling. (A) Immunoblot analysis of p-ERK1/2, p-p38, and p-SAPK/JNK MAPKs in WT, *Myd88* ^−/−^ *Trif* ^−/−^, and *Myd88* ^−/−^ *Rip2* ^−/−^ macrophages infected with WT or Δ*dotA L. pneumophila* at an MOI = 50. Total p38 MAPK is shown as a loading control. Data are representative of at least three independent experiments. (B) Immunoblot analysis of p-p38 and p-SAPK/JNK MAPKs in *Myd88* ^−/−^ *Rip2* ^−/−^ macrophages infected with WT, Δ*dotA*, or Δ*flaA L. pneumophila* at an MOI = 50. Total p38 MAPK is shown as a loading control. Data are representative of at least two independent experiments. (C) Immunoblot analysis of p-MKK4, p-MKK3/6, p-p38, p-SAPK/JNK, and p-c-Jun in *Myd88* ^−/−^ *Rip2* ^−/−^ macrophages infected with WT or Δ*dotA L. pneumophila* at an MOI = 50. Total p38 MAPK is shown as a loading control. Data are representative of at least two independent experiments. (D) Immunoblot analysis of p-p38 and p-SAPK/JNK in *Myd88* ^−/−^ macrophages infected with WT, Δ*dotA*, or Δ*icmS L. pneumophila* at an MOI = 50. Total p38 MAPK is shown as a loading control. Data are representative of at least three independent experiments. (E) Quantitative RT-PCR analysis of *Myd88* ^−/−^ *Rip2^−/−^* macrophages infected with WT, Δ*dotA*, or Δ*icmS L. pneumophila* at an MOI = 25 for four hours. Data represent the mean fold induction±SEM relative to uninfected macrophages of the assay performed in triplicate and are representative of at least three independent experiments. (F) Immunoblot analysis of p-p38 and p-SAPK/JNK in *Myd88* ^−/−^ *Rip2* ^−/−^ macrophages pretreated with or without chloramphenicol (25 µg/mL) for 30 minutes prior to infection with WT or Δ*dotA L. pneumophila* at an MOI = 50. Total p38 is shown as a loading control.

Microarray analysis and qPCR data revealed robust T4SS-dependent transcription of dual specificity phosphatase 1 (*Dusp1*) as well as other *Dusp* genes (Figure 4C and data not shown). Dusps are known to downregulate MAPK signaling. This indicated there was host MAPK activation in response to the T4SS. Therefore, we examined the activation state of the three canonical MAPK pathways, ERK1/2, p38, and SAPK/JNK, in response to *L. pneumophila*. In WT macrophages, there was robust and rapid activation of all three MAPKs in response to both WT and Δ*dotA L. pneumophila* infection (Figure 5A). In contrast, in *Myd88* ^−/−^ *Trif* ^−/−^ macrophages, there was ERK1/2 activation in response to both WT and Δ*dotA* mutant *L. pneumophila*, but p38 and SAPK/JNK MAPK activation was observed only in response to WT *L. pneumophila* (Figure 5A). This indicates that ERK1/2 activation is MyD88- and T4SS-independent. In contrast, p38 and SAPK/JNK MAPK signaling in response to *L. pneumophila* can be dissected into a MyD88-dependent, T4SS-independent pathway and a MyD88-independent, T4SS-dependent pathway.

Nod1 and Nod2 signaling through RIP2 also activate the p38 and SAPK/JNK MAPK pathways [10],[53],[54]. However, T4SS-dependent p38 and SAPK/JNK MAPK activation was RIP2-independent (Figure 5A), and T4SS-dependent MAPK activation was also observed in *Nod1* ^−/−^ *Nod2* ^−/−^ macrophages (data not shown). MAPK activation was also flagellin-independent (Figure 5B). T4SS-dependent MKK3/6 and MKK4 activation was observed, as well as activation of the transcription factors c-Jun and ATF2 (Figure 5C and data not shown). This suggests that a T4SS-dependent stimulus upstream of MKK3/6 and MKK4 is responsible for p38 and SAPK/JNK MAPK activation.

Treatment of cells with bacterial pore-forming toxins such as streptolysin O activates p38 MAPK by an unknown mechanism [55],[56]. Therefore, we considered whether p38 and SAPK/JNK MAPK activation was due to T4SS-mediated pore formation in the host membrane or alternatively required sustained T4SS translocation into the host cell. To address this, we examined Δ*icmS* and Δ*icmW* mutant *L. pneumophila*. IcmS and IcmW form a T4SS chaperone complex that is not essential for T4SS function per se, but the IcmSW complex is required for the efficient translocation of a large subset of T4SS effectors needed for formation of the ER-derived vacuole in which *L. pneumophila* replicates [57]–[60]. Δ*icmS* or Δ*icmW* mutants retain T4SS-mediated pore formation in the host membrane, but vacuoles containing these mutants fuse with the endolysosomal pathway within a few minutes of uptake [57],[61]. p38 and SAPK/JNK MAPK activation was undetectable in macrophages infected with the Δ*icmS* or Δ*icmW* mutant (Figure 5D and data not shown), indicating that pore formation alone is insufficient for robust p38 and SAPK/JNK MAPK activation. Transcription of multiple genes, including *Il1a*, *Il1b*, and *Cox2*, was significantly decreased in *Myd88^−/−^* macrophages infected with the Δ*icmS* mutant compared to WT bacteria (Figure 5E). Genes such as *Ifnb* were still robustly transcribed (Figure 5E and data not shown), indicating IcmS-dependent and -independent gene transcription. Examination of cytokine production in WT macrophages revealed that although there was a slight decrease in response to the Δ*icmS* mutant, there was still more robust cytokine production compared to infection with the Δ*dotA* mutant (Figure S3). These data suggest a subset of the host responses require IcmS-dependent translocation of effector proteins, whereas additional host responses are activated by the T4SS by a process independent of IcmS function.

There was still robust p38 and SAPK/JNK MAPK activation in macrophages infected with *L. pneumophila* in the presence of chloramphenicol, which inhibits bacterial translation but not the T4SS (Figure 5F), indicating that *de novo* protein synthesis of a T4SS substrate was not required. However, many *L. pneumophila* T4SS substrates are synthesized prior to infection [62]. Therefore, we considered whether putative *L. pneumophila* T4SS effectors had the potential to activate MAPK signaling. The *L. pneumophila* genome contains three genes encoding putative Ser/Thr protein kinases unique to *L. pneumophila* and absent from nonpathogenic bacteria: *legK1*, *legK2*, and *legK3* [63],[64]. LegK1, LegK2, and LegK3 were efficiently translocated into host cells by the T4SS (Figure S4A). However, T4SS-dependent p38 phosphorylation was not affected in *Myd88* ^−/−^ *Rip2* ^−/−^ macrophages infected with the Δ*legK1*, Δ*legK2*, Δ*legK3 L. pneumophila* triple mutant, indicating that these three genes are not required for MAPK activation (Figure S4B). Taken together, these data indicate that MAPK activation requires a fully functional T4SS and is either a direct or indirect response to translocated bacterial proteins.

### p38 and SAPK/JNK MAPK signaling is required for optimal T4SS-dependent gene expression

Treatment of *Myd88* ^−/−^ *Rip2* ^−/−^ macrophages with the p38 MAPK inhibitor SB202190 or the SAPK/JNK MAPK inhibitor JNK II prior to *L. pneumophila* infection revealed that full T4SS-dependent transcription of several genes required p38 and SAPK/JNK MAPK signaling (Figure S5). SB202190 and JNK II treatment did not affect *L. pneumophila* host cell entry or intracellular replication (data not shown). Therefore, p38 and SAPK/JNK MAPK signaling contributes to MyD88-independent, Dot/Icm-dependent gene transcription in response to *L. pneumophila* infection. Treatment of WT macrophages with SB202190 prior to bacterial infection also inhibited cytokine gene transcription (data not shown), as optimal cytokine transcription requires TLR-dependent p38 MAPK signaling [65],[66].

**Figure 6:**
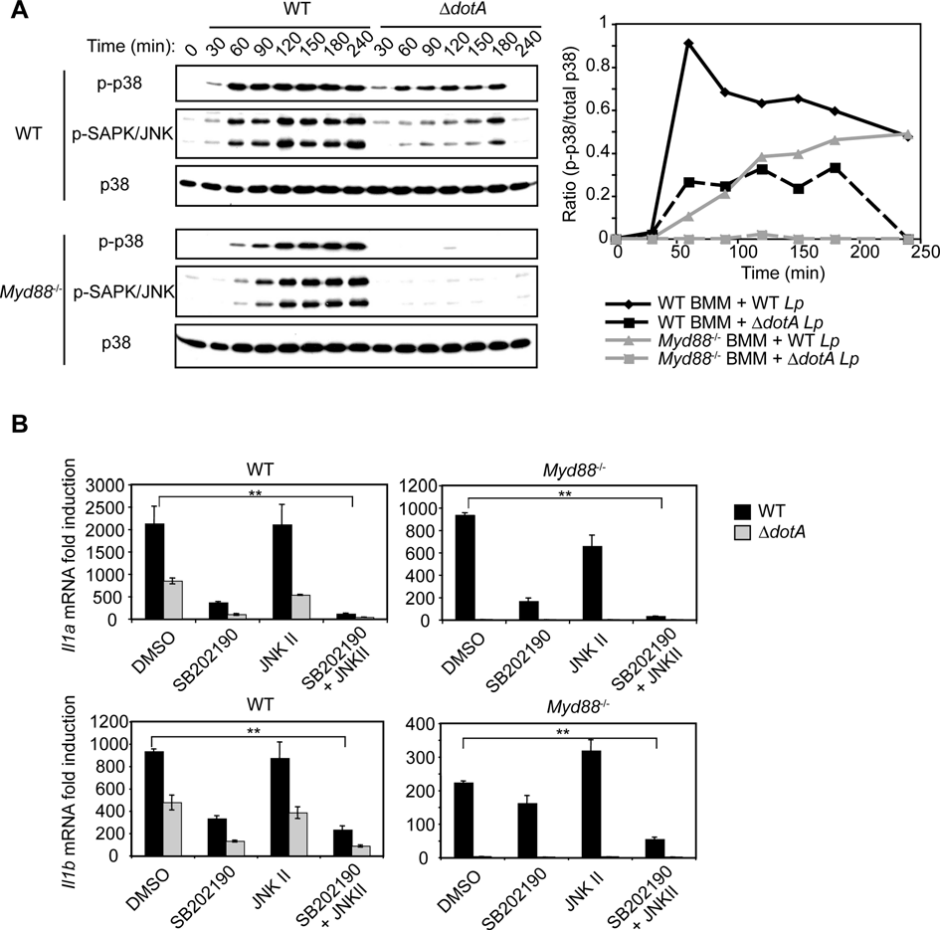
MyD88-dependent and T4SS-dependent p38 and SAPK/JNK MAPK signaling collaborate to induce a maximal transcriptional response. (A) Immunoblot analysis of p-p38 and p-SAPK/JNK MAPKs in WT and *Myd88^−/−^* macrophages infected with WT and Δ*dotA L. pneumophila* at an MOI = 50. Total p38 is shown as a loading control. Shown on the right is a graphical representation of of the immunoblot analysis depicting the ratio of p-p38 intensity to total p38 intensity versus time. Immunoblots were quantified using ImageJ. Data are representative of at least two independent experiments. (B) Quantitative RT-PCR analysis of WT and *Myd88* ^−/−^ macrophages. Macrophages were first infected with WT and Δ*dotA* mutant bacteria at an MOI = 25, then treated two hours later with 10 µM SB202190, 10 µM JNK II, 10 µM SB202190 plus 10 µM JNK II, or an equal volume of DMSO (vehicle control), and RNA harvested four hours after infection. Data represented the mean fold induction±SEM relative to uninfected cells of the assay performed in triplicate and are representative of at least two independent experiments.

A detailed analysis of the temporal kinetics of p38 and SAPK/JNK MAPK signaling revealed that WT macrophages infected with WT and Δ*dotA* mutant *L. pneumophila* infection activate MAPKs within 30 minutes (Figure 6A). MAPK activation in response to WT *L. pneumophila* peaked by 60 minutes and was sustained for at least 4 hours, whereas MAPK activation in response to the Δ*dotA* mutant was decreased and undetectable by 4 hours (Figure 6A). In contrast, in *Myd88* ^−/−^ macrophages, MAPK activation in response to WT *L. pneumophila* was delayed, detectable at 60 minutes (Figure 5A and 6A), peaked at 120 minutes, and sustained for at least 4 hours post infection (Figure 6A). This indicates that the increased and sustained MAPK activation in WT macrophages infected with WT *L. pneumophila* represents the aggregate of T4SS-independent, MyD88-dependent MAPK activation and T4SS-dependent, MyD88-independent MAPK activation (Figure 6A). Inhibitors were used to determine if this aggregate signal was responsible for the increased cytokine transcription seen in WT macrophages infected with WT *L. pneumophila*. The p38 MAPK inhibitor SB202190, the SAPK/JNK MAPK inhibitor JNK II, or both were added to *Myd88* ^−/−^ or WT macrophages at 120 minutes post-infection, the time of peak MyD88-independent, T4SS-dependent MAPK activation. *Il1a* and *Il1b* transcription was measured four hours post-infection. *Il1a* and *Il1b* transcription was decreased in *Myd88* ^−/−^ and WT macrophages treated singly with p38 or SAPK/JNK MAPK inhibitors, with a synergistic decrease observed when both p38 and SAPK/JNK MAPKs were inhibited. This demonstrates that sustained MAPK activation in response to the T4SS is critical for optimal cytokine transcription during *L. pneumophila* infection.

## Discussion

Our data support a model in which coincident detection of multiple bacterial components and the integration of these signals enables immune discrimination of pathogenic and nonpathogenic microbes. This results in either a full proinflammatory response against a pathogen or a tempered immune response to an avirulent microbe. We show here that the robust innate immune response to virulent *L. pneumophila* requires synergy between TLR-dependent detection of *L. pneumophila* PAMPs and TLR-independent detection of *L. pneumophila* T4SS-translocated bacterial factors in the host cytosol. These data identify a T4SS-dependent and RIP2-independent p38 and SAPK/JNK MAPK pathway that synergizes with MyD88-dependent MAPK activation for optimum cytokine transcription.

IL-1β secretion in response to *L. pneumophila* infection exemplifies this model of multifactorial control of immune responses against a pathogen. Data shown here indicate that in addition to TLR signaling, T4SS detection is also required for optimal pro-IL-1β expression in response to *L. pneumophila* infection. The third and final step involving inflammasome activation and caspase-1-dependent IL-1β processing and secretion requires detection of the T4SS and flagellin [18],[21]. This demonstrates that at least three different signaling pathways, one that is TLR-dependent and two that are TLR-independent and T4SS-dependent, are required for IL-1β production and secretion.

p38 and SAPK/JNK MAPK activation in response to *L. pneumophila* was dissected into two distinct pathways with differing temporal kinetics and signaling intensity: 1) temporally rapid TLR-dependent MAPK activation and 2) temporally delayed and sustained TLR-independent, RIP2-independent, T4SS-dependent MAPK activation. This demonstrates that there are two distinct modes of MAPK activity that are differentially regulated in a temporal manner and synergize to result in increased cytokine expression in response to *L. pneumophila*. This explains a previous observation that WT macrophages infected with WT *L. pneumophila* exhibited increased SAPK/JNK MAPK activation compared to those infected with the Δ*dotA* mutant [67]. It is still unclear, however, what aspect of the T4SS is responsible for the MyD88- and RIP2-independent p38 and SAPK/JNK MAPK activation. *L. pneumophila* lacking the T4SS chaperone IcmS do not induce detectable p38 and SAPK/JNK activation. This indicates that initial pore formation induced in the host cell membrane by the T4SS is insufficient for robust MAPK activation. Instead, MAPK activation may require the detection of an IcmS-guided T4SS substrate or sustained translocation to achieve a minimal substrate concentration required for detection. This T4SS substrate could be a PAMP such as a cell wall component.

Another possibility is that subversion of host cell pathways important for remodeling of the *L. pneumophila*-containing vacuole into an ER-derived organelle could potentially activate p38 and SAPK/JNK MAPKs. It remains possible that a *L. pneumophila* T4SS effector directly modulates p38 and SAPK/JNK MAPK signaling or that the cell senses the biochemical activity of the effector. This possibility led us to test whether three *L. pneumophila* putative Ser/Thr protein kinases predicted to be translocated by the T4SS into host cells were important for MAPK activation. Indeed, the three *L. pneumophila* LegK proteins were found to be type IV substrates; however, a *L. pneumophila* mutant deficient in all three *legK* genes still induced p38 and SAPK/JNK MAPK activation, indicating that the LegK proteins are not required for the T4SS MAPK response. Thus, the possibility exists that multiple effectors with different biochemical activities could independently activate MAPK signaling, which would not be surprising given that this T4SS manipulates a variety of different cellular pathways using a repertoire of over 100 different effector proteins.

Immune pathways other than MAPK signaling were also activated by the *L. pneumophila* T4SS. For example, consistent with the defect in p38 and SAPK/JNK MAPK activation, *Myd88^−/−^Rip2^−/−^* macrophages infected with the Δ*icmS* mutant displayed impaired transcriptional induction of several genes, including *Il1a* and *Il1b*. However, the Δ*icmS* mutant still induced robust *Ifnb* transcription, indicating differing signaling requirements for the transcription of *Ifnb* compared to *Il1a* and *Il1b*. Interestingly, in WT macrophages, the Δ*icmS* mutant still stimulated more cytokine production than the Δ*dotA* mutant, indicating that additional T4SS-dependent pathways other than p38 and SAPK/JNK MAPKs also contribute to cytokine production.

Microarray analysis revealed a large number of genes induced by T4S independently of MyD88 and RIP2 signaling. Although our analysis focused on MAPK signaling, other MyD88- and RIP2-independent signaling pathways are also likely to respond to the T4SS. *L. pneumophila* T4S induces *Ifnb* transcription in host cells [25],[26]. It has been suggested that DNA translocated by the *L. pneumophila* T4SS triggers the ISD response, which leads to *Ifnb* transcription [25]. Comparison of the ISD response with the *L. pneumophila* T4SS response revealed overlapping yet distinct transcriptional programs. The ISD response differs from the T4SS MAPK response, as the ISD response does not involve NF-κB or MAPK signaling and instead requires IRF3. Additionally, infection with the *L. pneumophila* Δ*icmS* mutant induces robust *Ifnb* transcription with no detectable MAPK activation. This indicates that the MyD88- and RIP2-independent T4SS response is comprised of several discrete pathways, including MAPK signaling and the IRF3-dependent *Ifnb* response. Identification of other signaling pathways responsible for T4SS-dependent, MyD88- and RIP2-independent gene expression will elucidate other mechanisms of innate immune discrimination of pathogens.

Using mice either singly or doubly deficient for MyD88 and RIP2, we showed that NF-κB activation in response to *L. pneumophila* is controlled by two distinct pathways: 1) MyD88-dependent, T4SS-independent signaling and 2) MyD88-independent, RIP2-dependent, and T4SS-dependent signaling. This indicates that TLR-independent, T4SS-dependent NF-κB activation is controlled by Nod1 and Nod2 detection of a T4SS substrate, possibly peptidoglycan, in the host cytosol. Previous observations showed that infection with a low dose of *L. pneumophila* allowed for the detection of T4SS-dependent NF-κB activation in macrophages lacking either MyD88 or Nod1 [52]. Since pharmacological inhibition of NF-κB inhibited bacterial replication and host cell viability, it was proposed that a T4SS effector modulates NF-κB signaling to promote host cell survival through the upregulation of anti-apoptotic factors [52]. Our data indicate that NF-κB activation in the absence of both MyD88 and RIP2 is undetectable; however, *L. pneumophila* were able to replicate similarly in *Myd88^−/−^Rip2^−/−^* macrophages and control heterozygous macrophages (data not shown), suggesting that NF-κB activation is not essential for host cell survival during *L. pneumophila* infection. Because anti-apoptotic genes are also regulated by p38 MAPK signaling [68], it is likely that the T4SS-dependent MAPK activation described here is sufficient to upregulate host cell survival functions in the absence of NF-κB activity.

In conclusion, we have described multiple TLR-dependent and -independent signaling pathways triggered by *L. pneumophila* and its T4SS. In particular, we have demonstrated that the T4SS induces MyD88- and RIP2-independent p38 and SAPK/JNK MAPK activation. This T4SS-dependent MAPK signaling synergizes with MyD88- and RIP2-dependent signaling, leading to increased immune gene expression. Further identification of the host signaling pathways that comprise the T4SS response will elucidate how *L. pneumophila* manipulates host signaling pathways as well as how the innate immune system detects bacterial secretion systems and initiates immunity against pathogens.

## Materials and Methods

### Bacterial strains and reagents

*Legionella pneumophila* serogroup 1 strains were used. When indicated in the figure legends or text, mice and macrophages were infected with Lp02 (CR24; *thyA*), a thymidine auxotroph derived from strain Lp01 [31] or the isogenic mutant strains CR25 (Δ*dotA*, *thyA*), CR1665 (Δ*flaA*, *thyA*), and SS29 (Δ*dotA*, Δ*icmS*, and *thyA*). Otherwise, macrophages were infected with Lp01 (CR39; WT) or the isogenic mutant strains CR393 (Δ*icmS*), CR1668 (Δ*flaA*), or CR58 (Δ*dotA*) [61]. For *in vitro* studies, *L. pneumophila* were cultured for two days on charcoal yeast extract agar prior to infection. For *in vivo* studies, *L. pneumophila* were grown as described previously (Archer and Roy, 2006).

### Mice

C57BL/6 and A/J mice were purchased from Jackson Laboratories. *Myd88* ^−/−^ [51], *Rip2* ^−/−^ [11], *Nod1* ^−/−^ [6], *Nod2* ^−/−^ [10], *Nod1* ^−/−^ *Nod2* ^−/−^, and *Tlr2* ^−/−^ [69] mice have been described. *Myd88* ^−/−^ *Trif* ^−/−^ and *Myd88* ^−/−^ *Rip2* ^−/−^ mice were provided by R. Medzhitov. Animals were maintained in accordance with the guidelines of the Yale University Institutional Animal Use and Care Committee (protocol 07847).

### In vivo infection studies

In vivo infection studies utilized 8 week-old mice. Mice were anesthetized by subcutaneous injection of a 0.2 mL solution containing ketamine (12 mg/ml) and xylazine (1.2 mg/ml). Mice were infected intranasally with 40 µl of a bacterial suspension containing 5×10^6^ CFUs *L. pneumophila*. 24 h after infection, bronchoalveolar lavage fluid was harvested. Live animal experiments were approved by the Yale University Institutional Animal Care and Use Committee (protocol 07847).

### Macrophage infection conditions

Bone marrow-derived macrophages (BMMs) were cultured in RPMI containing 30% L cell supernatant, 20% FBS and replated one day prior to infection in RPMI containing 15% L cell supernatant, 10% FBS. For experiments involving cytokine production measurements by ELISA, BMMs in 24-well plates (2.5×10^5^ cells/well) were infected with *L. pneumophila* at an MOI = 5 for 24 hours. For experiments involving immunoblot analysis of infected cells, BMMs in 24-well plate (2.5×10^5^ cells/well) were infected with *L. pneumophila* at an MOI = 50 for timepoints ranging from 0 to 180 min. For experiments involving harvesting RNA from infected cells, BMMs in 24-well plates (2.5×10^5^ cells/well) were infected with *L. pneumophila* at an MOI = 25 for 4 hours. To assess the involvement of p38 or SAPK/JNK MAPK in gene transcription, 10 µM of the p38 MAPK inhibitor SB202190 (Calbiochem), 10 µM of the SAPK/JNK MAPK inhibitor (JNK II), or an equivalent volume of dimethyl sulfoxide (vehicle control) were added to BMMs one hour prior to infection or two hours post-infection. For all infections, bacteria were spun down onto the macrophages at 1,000 RPM for five minutes prior to incubation.

### RNA preparation and quantitative RT-PCR

For microarray analysis, BMMs were infected for four hours at an MOI = 25 with Lp02 or Lp03 and harvested into RNA Bee. Following isolation, RNA was cleaned using RNeasy Mini columns (Qiagen). For quantitative RT-PCR, RNA was isolated from infected BMMs, DNase-treated using the RNeasy Mini kit and RNase-free DNase set (Qiagen), and reverse transcribed with Superscript II (Invitrogen). Quantitative PCR was performed on a real-time detection system (iCycler; Bio-Rad Laboratories) using the iQ SYBR Green super mix (Bio-Rad). Gene mRNA abundance was normalized to HPRT mRNA abundance and compared to normalized gene mRNA abundance in uninfected cells using the ΔΔCT method to calculate fold induction. A list of gene-specific primers is in Table S1.

### Microarray analysis

Microarray analysis was performed using Affymetrix GeneChip Mouse Genome 430 2.0 arrays at the Yale University William M. Keck facility. The complete data set is available at the Gene Expression Omnibus (http://www.ncbi.nlm.nih.gov/geo) under accession number GSE13147. Lists of genes differentially regulated two-fold or more in response to WT *L. pneumophila* versus *dotA L. pneumophila* in *Myd88^−/−^Trif^−/−^* and *Myd88^−/−^Rip2^−/−^* macrophages are in Tables S2, S3, and S4.

### Immunoblotting

Infected BMMs were directly lysed in 1× SDS-PAGE sample buffer. Lysates were separated by SDS-PAGE and transferred to Immobilon P membranes (Millipore). Antibodies against IL-1β (BD Biosciences), p-p38 MAPK, p38 MAPK, p-SAPK/JNK, p-ERK1/2, p-MKK3/6, p-MKK4, p-c-Jun (Cell Signaling Technology), IκB (Santa Cruz Biotechnology), and actin (Sigma) were used.

### ELISA

Harvested supernatants from infected BMMs were analyzed using IL-1α, IL-6, IL-12 (BD Biosciences), KC, and TNF (R&D Systems) ELISA antibodies.

### Generation of Cya:gene plasmids and Cya translocation assay

*legK1*, *legK2*, and *legK3* were amplified from *L. pneumophila* genomic DNA using primers 29–34 described in Table S1. They were then cloned into pEC34 [60] to generate N-terminal Cya:gene fusions. The resulting plasmids, pMMB207NT.*cya* [62], and pMMB207NT.*ralF* [62] were transformed into WT or Δ*dotA* mutant *L. pneumophila*. The Cya assays were performed as previously described [62]. Briefly, 1×10^5^ CHO-FcγRII cells per well of a 24-well plate were infected with opsonized *L. pneumophila* expressing Cya fusion proteins at an MOI = 30. After 1 hour of infection at 37°C, cells were washed with PBS and lysed. Total cAMP was extracted and quantified using the cAMP Enzyme Immunoassay System (GE Healthcare).

### Generation of bacterial isogenic mutant strains

The in-frame *legK1*, *legK2*, and *legK3* deletions were constructed by amplifying 5′ and 3′ gene fragments and then joining them by recombinant PCR using primers 35–46 listed in Table S1. The recombinant PCR product was digested and ligated into the vector pSR47S. The *legK1*, *legK2*, and *legK3* deletions were introduced onto the chromosome of *L. pneumophila* strain Lp01 by allelic exchange as previously described [70].

## Supporting Information

Figure S1 Quantitation of IκBα degradation in *Myd88* ^−/−^ and *Myd88* ^−/−^ *Rip2* ^−/−^ macrophages infected with WT or Δ*dotA L. pneumophila*.
(2.29 MB TIF)

Figure S2 *L. pneumophila* infection induces Dot/Icm-dependent cytokine production in the absence of Nod1 and Nod2.
(3.95 MB TIF)

Figure S3 The *L. pneumophila* Δ*icmS* mutant induces slightly decreased cytokine production.
(3.03 MB TIF)

Figure S4 The *L. pneumophila* Dot/Icm system translocates three Ser/Thr protein kinases that are dispensable for p38 MAPK activation.
(2.36 MB TIF)

Figure S5 p38 and SAPK/JNK MAPK signaling contribute to Dot/Icm-dependent gene transcription in the absence of MyD88 and RIP2.
(1.83 MB TIF)

Table S1 Primers used in this study.
(0.04 MB XLS)

Table S2 Genes that exhibit two-fold or greater Dot/Icm-dependent transcriptional changes in both *Myd88* ^−/−^ *Trif* ^−/−^ and *Myd88* ^−/−^ *Rip2* ^−/−^ macrophages.
(0.51 MB XLS)

Table S3 Genes that exhibit two-fold or greater Dot/Icm-dependent transcriptional changes in *Myd88* ^−/−^ *Trif* ^−/−^ macrophages but not in *Myd88* ^−/−^ *Rip2* ^−/−^ macrophages.
(0.07 MB XLS)

Table S4 Genes that exhibit two-fold or greater Dot/Icm-dependent transcriptional changes in *Myd88* ^−/−^ *Rip2* ^−/−^ macrophages but not in *Myd88* ^−/−^ *Trif* ^−/−^ macrophages.
(0.71 MB XLS)

Table S5 Genes that exhibit two-fold or greater Dot/Icm-dependent transcriptional changes in both *Myd88* ^−/−^ *Trif* ^−/−^ and *Myd88* ^−/−^ *Rip2* ^−/−^ macrophages and are shared by the ISD response in *Myd88* ^−/−^ *Trif* ^−/−^ macrophages.
(0.05 MB XLS)

Table S6 Genes that exhibit two-fold or greater Dot/Icm-dependent transcriptional changes in both *Myd88* ^−/−^ *Trif* ^−/−^ and *Myd88* ^−/−^ *Rip2* ^−/−^ macrophages and are unique from the ISD response in *Myd88* ^−/−^ *Trif* ^−/−^ macrophages.
(0.08 MB XLS)

## References

[1] Janeway CA (1989). Approaching the asymptote? Evolution and revolution in immunology.. Cold Spring Harb Symp Quant Biol.

[2] Bhavsar AP, Guttman JA, Finlay BB (2007). Manipulation of host-cell pathways by bacterial pathogens.. Nature.

[3] Inohara, Chamaillard, McDonald C, Nunez G (2005). NOD-LRR proteins: role in host-microbial interactions and inflammatory disease.. Annu Rev Biochem.

[4] Ting JP, Kastner DL, Hoffman HM (2006). CATERPILLERs, pyrin and hereditary immunological disorders.. Nat Rev Immunol.

[5] Fritz JH, Ferrero RL, Philpott DJ, Girardin SE (2006). Nod-like proteins in immunity, inflammation and disease.. Nat Immunol.

[6] Chamaillard M, Hashimoto M, Horie Y, Masumoto J, Qiu S (2003). An essential role for NOD1 in host recognition of bacterial peptidoglycan containing diaminopimelic acid.. Nat Immunol.

[7] Girardin SE, Boneca IG, Viala J, Chamaillard M, Labigne A (2003). Nod2 is a general sensor of peptidoglycan through muramyl dipeptide (MDP) detection.. J Biol Chem.

[8] Girardin SE, Boneca IG, Carneiro LA, Antignac A, Jehanno M (2003). Nod1 detects a unique muropeptide from gram-negative bacterial peptidoglycan.. Science.

[9] Inohara N, Ogura Y, Fontalba A, Gutierrez O, Pons F (2003). Host recognition of bacterial muramyl dipeptide mediated through NOD2. Implications for Crohn's disease.. J Biol Chem.

[10] Kobayashi KS, Chamaillard M, Ogura Y, Henegariu O, Inohara N (2005). Nod2-dependent regulation of innate and adaptive immunity in the intestinal tract.. Science.

[11] Kobayashi K, Inohara N, Hernandez LD, Galan JE, Nunez G (2002). RICK/Rip2/CARDIAK mediates signalling for receptors of the innate and adaptive immune systems.. Nature.

[12] Chin AI, Dempsey PW, Bruhn K, Miller JF, Xu Y (2002). Involvement of receptor-interacting protein 2 in innate and adaptive immune responses.. Nature.

[13] Hsu YM, Zhang Y, You Y, Wang D, Li H (2007). The adaptor protein CARD9 is required for innate immune responses to intracellular pathogens.. Nat Immunol.

[14] Petrilli V, Dostert C, Muruve DA, Tschopp J (2007). The inflammasome: a danger sensing complex triggering innate immunity.. Curr Opin Immunol.

[15] Sutterwala FS, Ogura Y, Flavell RA (2007). The inflammasome in pathogen recognition and inflammation.. J Leukoc Biol.

[16] Miao EA, Alpuche-Aranda CM, Dors M, Clark AE, Bader MW (2006). Cytoplasmic flagellin activates caspase-1 and secretion of interleukin 1beta via Ipaf.. Nat Immunol.

[17] Franchi L, Amer A, Body-Malapel M, Kanneganti TD, Ozoren N (2006). Cytosolic flagellin requires Ipaf for activation of caspase-1 and interleukin 1beta in salmonella-infected macrophages.. Nat Immunol.

[18] Zamboni DS, Kobayashi KS, Kohlsdorf T, Ogura Y, Long EM (2006). The Birc1e cytosolic pattern-recognition receptor contributes to the detection and control of *Legionella pneumophila* infection.. Nat Immunol.

[19] Molofsky AB, Byrne BG, Whitfield NN, Madigan CA, Fuse ET (2006). Cytosolic recognition of flagellin by mouse macrophages restricts Legionella pneumophila infection.. J Exp Med.

[20] Ren T, Zamboni DS, Roy CR, Dietrich WF, Vance RE (2006). Flagellin-deficient Legionella mutants evade caspase-1- and Naip5-mediated macrophage immunity.. PLoS Pathog.

[21] Lightfield KL, Persson J, Brubaker SW, Witte CE, von Moltke J (2008). Critical function for Naip5 in inflammasome activation by a conserved carboxy-terminal domain of flagellin.. Nat Immunol.

[22] O'Riordan M, Yi CH, Gonzales R, Lee KD, Portnoy DA (2002). Innate recognition of bacteria by a macrophage cytosolic surveillance pathway.. Proc Natl Acad Sci U S A.

[23] Stockinger S, Materna T, Stoiber D, Bayr L, Steinborn R (2002). Production of type I IFN sensitizes macrophages to cell death induced by Listeria monocytogenes.. J Immunol.

[24] McCaffrey RL, Fawcett P, O'Riordan M, Lee KD, Havell EA (2004). A specific gene expression program triggered by Gram-positive bacteria in the cytosol.. Proc Natl Acad Sci U S A.

[25] Stetson DB, Medzhitov R (2006). Recognition of cytosolic DNA activates an IRF3-dependent innate immune response.. Immunity.

[26] Opitz B, Vinzing M, van Laak V, Schmeck B, Heine G (2006). *Legionella pneumophila* induces IFNbeta in lung epithelial cells via IPS-1 and IRF3, which also control bacterial replication.. J Biol Chem.

[27] Leber JH, Crimmins GT, Raghavan S, Meyer-Morse NP, Cox JS (2008). Distinct TLR- and NLR-mediated transcriptional responses to an intracellular pathogen.. PLoS Pathog.

[28] McDade JE, Shepard CC, Fraser DW, Tsai TR, Redus MA (1977). Legionnaires' disease: isolation of a bacterium and demonstration of its role in other respiratory disease.. N Engl J Med.

[29] Shin S, Roy CR (2008). Host cell processes that influence the intracellular survival of *Legionella pneumophila*.. Cell Microbiol.

[30] Marra A, Blander SJ, Horwitz MA, Shuman HA (1992). Identification of a *Legionella pneumophila* locus required for intracellular multiplication in human macrophages.. Proc Natl Acad Sci U S A.

[31] Berger KH, Isberg RR (1993). Two distinct defects in intracellular growth complemented by a single genetic locus in *Legionella pneumophila*.. Mol Microbiol.

[32] Segal G, Purcell M, Shuman HA (1998). Host cell killing and bacterial conjugation require overlapping sets of genes within a 22-kb region of the *Legionella pneumophila* genome.. Proc Natl Acad Sci U S A.

[33] Vogel JP, Andrews HL, Wong SK, Isberg RR (1998). Conjugative transfer by the virulence system of *Legionella pneumophila*.. Science.

[34] Ninio S, Roy CR (2007). Effector proteins translocated by *Legionella pneumophila*: Strength in numbers.. Trends Microbiol.

[35] Roy CR, Berger KH, Isberg RR (1998). *Legionella pneumophila* DotA protein is required for early phagosome trafficking decisions that occur within minutes of bacterial uptake.. Mol Microbiol.

[36] Archer KA, Roy CR (2006). MyD88-dependent responses involving toll-like receptor 2 are important for protection and clearance of *Legionella pneumophila* in a mouse model of Legionnaires' disease.. Infect Immun.

[37] Hawn TR, Smith KD, Aderem A, Skerrett SJ (2006). Myeloid differentiation primary response gene (88)- and toll-like receptor 2-deficient mice are susceptible to infection with aerosolized *Legionella pneumophila*.. J Infect Dis.

[38] Amer A, Franchi L, Kanneganti TD, Body-Malapel M, Ozoren N (2006). Regulation of Legionella phagosome maturation and infection through flagellin and host Ipaf.. J Biol Chem.

[39] McHugh SL, Yamamoto Y, Klein TW, Friedman H (2000). Murine macrophages differentially produce proinflammatory cytokines after infection with virulent vs. avirulent *Legionella pneumophila*.. J Leukoc Biol.

[40] Neild AL, Roy CR (2003). Legionella reveal dendritic cell functions that facilitate selection of antigens for MHC class II presentation.. Immunity.

[41] Sporri R, Joller N, Albers U, Hilbi H, Oxenius A (2006). MyD88-dependent IFN-gamma production by NK cells is key for control of *Legionella pneumophila* infection.. J Immunol.

[42] Mintz CS, Chen JX, Shuman HA (1988). Isolation and characterization of auxotrophic mutants of Legionella pneumophila that fail to multiply in human monocytes.. Infect Immun.

[43] Diez E, Lee SH, Gauthier S, Yaraghi Z, Tremblay M (2003). Birc1e is the gene within the Lgn1 locus associated with resistance to Legionella pneumophila.. Nat Genet.

[44] Wright EK, Goodart SA, Growney JD, Hadinoto V, Endrizzi MG (2003). Naip5 affects host susceptibility to the intracellular pathogen Legionella pneumophila.. Curr Biol.

[45] Yamamoto Y, Klein TW, Newton CA, Widen R, Friedman H (1988). Growth of Legionella pneumophila in thioglycolate-elicited peritoneal macrophages from A/J mice.. Infect Immun.

[46] Kanneganti TD, Lamkanfi M, Nunez G (2007). Intracellular NOD-like receptors in host defense and disease.. Immunity.

[47] Wilmanski JM, Petnicki-Ocwieja T, Kobayashi KS (2007). NLR proteins: integral members of innate immunity and mediators of inflammatory diseases.. J Leukoc Biol.

[48] Viala J, Chaput C, Boneca IG, Cardona A, Girardin SE (2004). Nod1 responds to peptidoglycan delivered by the Helicobacter pylori cag pathogenicity island.. Nat Immunol.

[49] Fritz JH, Girardin SE, Fitting C, Werts C, Mengin-Lecreulx D (2005). Synergistic stimulation of human monocytes and dendritic cells by Toll-like receptor 4 and NOD1- and NOD2-activating agonists.. Eur J Immunol.

[50] Tada H, Aiba S, Shibata K, Ohteki T, Takada H (2005). Synergistic effect of Nod1 and Nod2 agonists with toll-like receptor agonists on human dendritic cells to generate interleukin-12 and T helper type 1 cells.. Infect Immun.

[51] Adachi O, Kawai T, Takeda K, Matsumoto M, Tsutsui H (1998). Targeted disruption of the MyD88 gene results in loss of IL-1- and IL-18-mediated function.. Immunity.

[52] Losick VP, Isberg RR (2006). NF-kappaB translocation prevents host cell death after low-dose challenge by Legionella pneumophila.. J Exp Med.

[53] Girardin SE, Tournebize R, Mavris M, Page AL, Li X (2001). CARD4/Nod1 mediates NF-kappaB and JNK activation by invasive Shigella flexneri.. EMBO Rep.

[54] Park JH, Kim YG, McDonald C, Kanneganti TD, Hasegawa M (2007). RICK/RIP2 mediates innate immune responses induced through Nod1 and Nod2 but not TLRs.. J Immunol.

[55] Ratner AJ, Hippe KR, Aguilar JL, Bender MH, Nelson AL (2006). Epithelial cells are sensitive detectors of bacterial pore-forming toxins.. J Biol Chem.

[56] Huffman DL, Abrami L, Sasik R, Corbeil J, van der Goot FG (2004). Mitogen-activated protein kinase pathways defend against bacterial pore-forming toxins.. Proc Natl Acad Sci U S A.

[57] Coers J, Kagan JC, Matthews M, Nagai H, Zuckman DM (2000). Identification of Icm protein complexes that play distinct roles in the biogenesis of an organelle permissive for Legionella pneumophila intracellular growth.. Mol Microbiol.

[58] Ninio S, Zuckman-Cholon DM, Cambronne ED, Roy CR (2005). The Legionella IcmS-IcmW protein complex is important for Dot/Icm-mediated protein translocation.. Mol Microbiol.

[59] Bardill JP, Miller JL, Vogel JP (2005). IcmS-dependent translocation of SdeA into macrophages by the *Legionella pneumophila* type IV secretion system.. Mol Microbiol.

[60] Cambronne ED, Roy CR (2007). The *Legionella pneumophila* IcmSW Complex Interacts with Multiple Dot/Icm Effectors to Facilitate Type IV Translocation.. PLoS Pathog.

[61] Zuckman DM, Hung JB, Roy CR (1999). Pore-forming activity is not sufficient for *Legionella pneumophila* phagosome trafficking and intracellular growth.. Mol Microbiol.

[62] Nagai H, Cambronne ED, Kagan JC, Amor JC, Kahn RA (2005). A C-terminal translocation signal required for Dot/Icm-dependent delivery of the Legionella RalF protein to host cells.. Proc Natl Acad Sci U S A.

[63] Chien M, Morozova I, Shi S, Sheng H, Chen J (2004). The genomic sequence of the accidental pathogen *Legionella pneumophila*.. Science.

[64] de Felipe KS, Pampou S, Jovanovic OS, Pericone CD, Ye SF (2005). Evidence for acquisition of Legionella type IV secretion substrates via interdomain horizontal gene transfer.. J Bacteriol.

[65] Park JM, Greten FR, Li ZW, Karin M (2002). Macrophage apoptosis by anthrax lethal factor through p38 MAP kinase inhibition.. Science.

[66] Akira S, Uematsu S, Takeuchi O (2006). Pathogen recognition and innate immunity.. Cell.

[67] Welsh CT, Summersgill JT, Miller RD (2004). Increases in c-Jun N-terminal kinase/stress-activated protein kinase and p38 activity in monocyte-derived macrophages following the uptake of *Legionella pneumophila*.. Infect Immun.

[68] Park JM, Greten FR, Wong A, Westrick RJ, Arthur JS (2005). Signaling pathways and genes that inhibit pathogen-induced macrophage apoptosis–CREB and NF-kappaB as key regulators.. Immunity.

[69] Takeuchi O, Hoshino K, Kawai T, Sanjo H, Takada H (1999). Differential roles of TLR2 and TLR4 in recognition of gram-negative and gram-positive bacterial cell wall components.. Immunity.

[70] Wiater LA, Sadosky AB, Shuman HA (1994). Mutagenesis of *Legionella pneumophila* using Tn903 dlllacZ: identification of a growth-phase-regulated pigmentation gene.. Mol Microbiol.

