# Supplementary figures and images for: Type IV Secretion-Dependent Activation of Host MAP Kinases Induces an Increased Proinflammatory Cytokine Response to Legionella pneumophila
Source: PLoS Pathog. 2008 Nov 28;4(11):e1000220. doi: 10.1371/journal.ppat.1000220 (PMC2582680; doi:10.1371/journal.ppat.1000220)

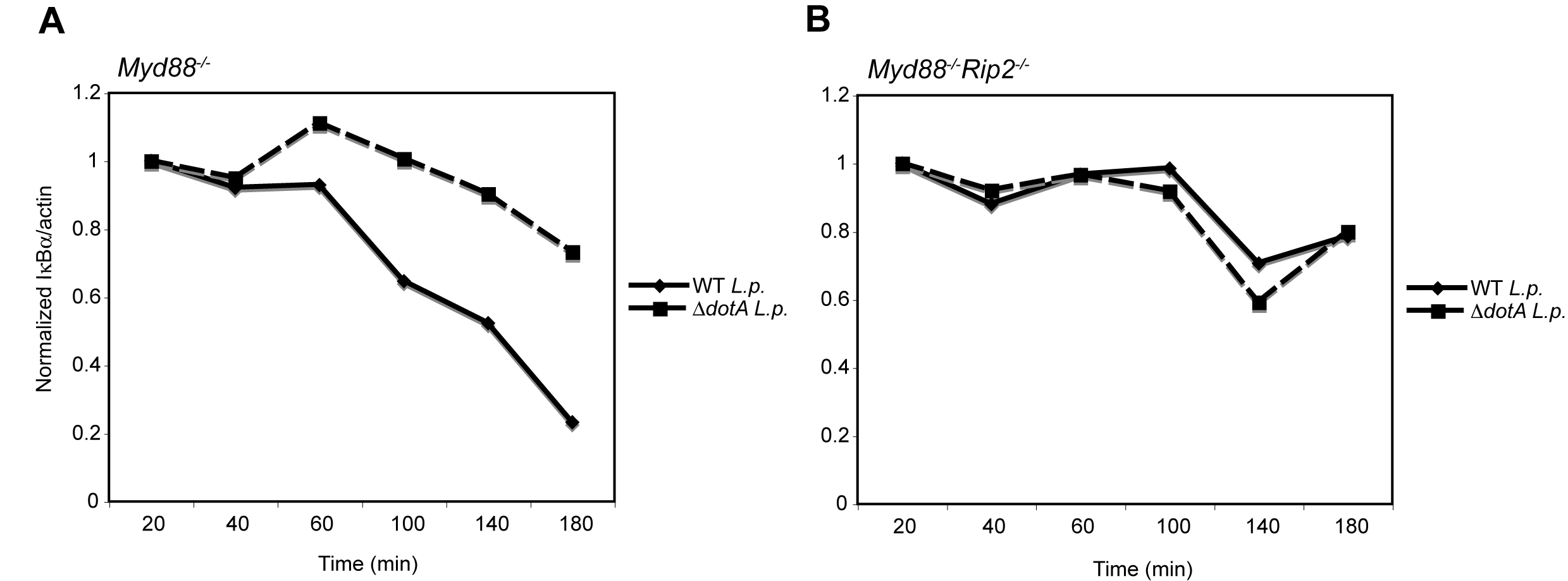

Supplement: Figure S1 — Quantitation of IκBα degradation in Myd88 −/− and Myd88 −/− Rip2 −/− macrophages infected with WT or ΔdotA L. pneumophila. (2.29 MB TIF) [file ppat.1000220.s001.tif]

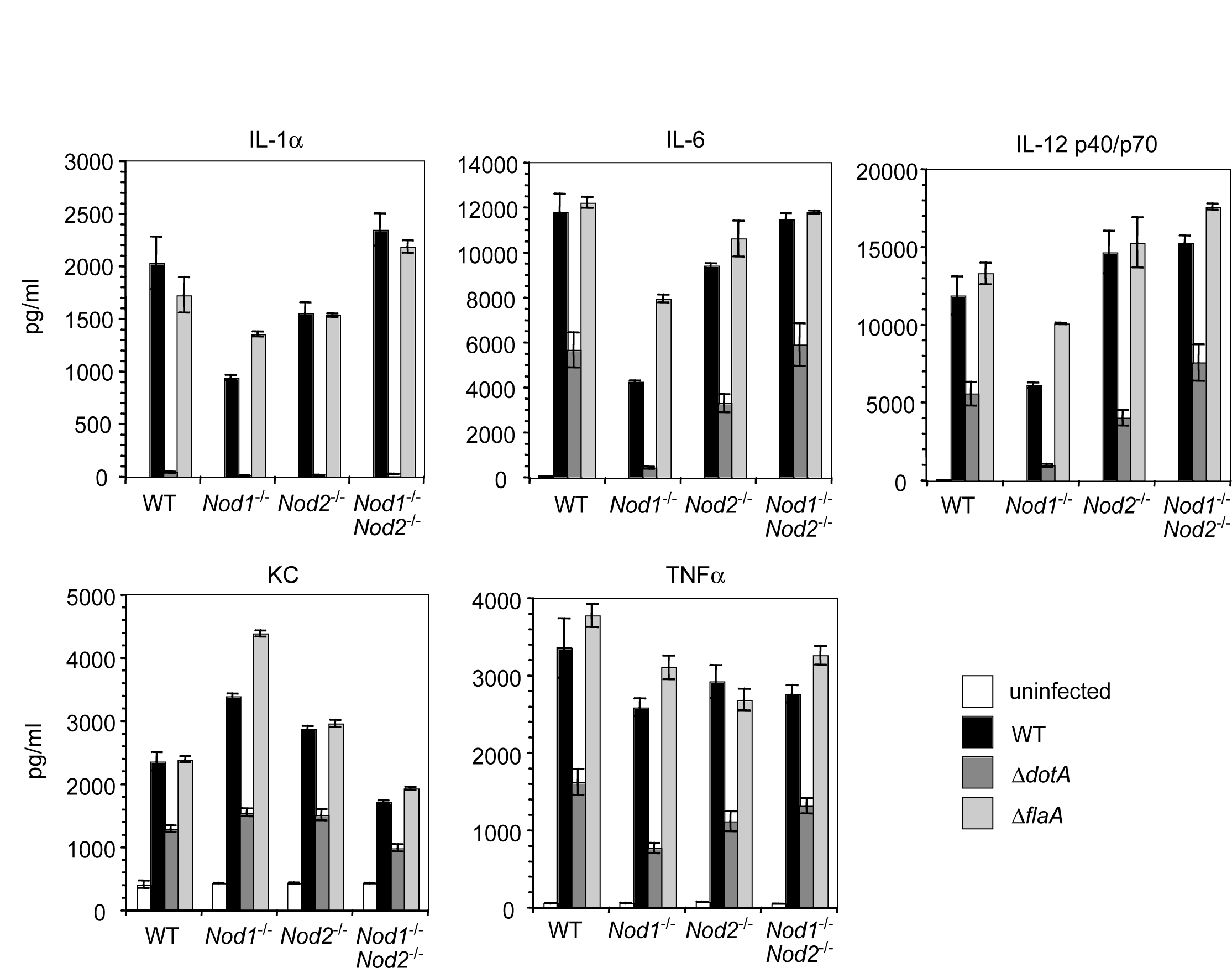

Supplement: Figure S2 — L. pneumophila infection induces Dot/Icm-dependent cytokine production in the absence of Nod1 and Nod2. (3.95 MB TIF) [file ppat.1000220.s002.tif]

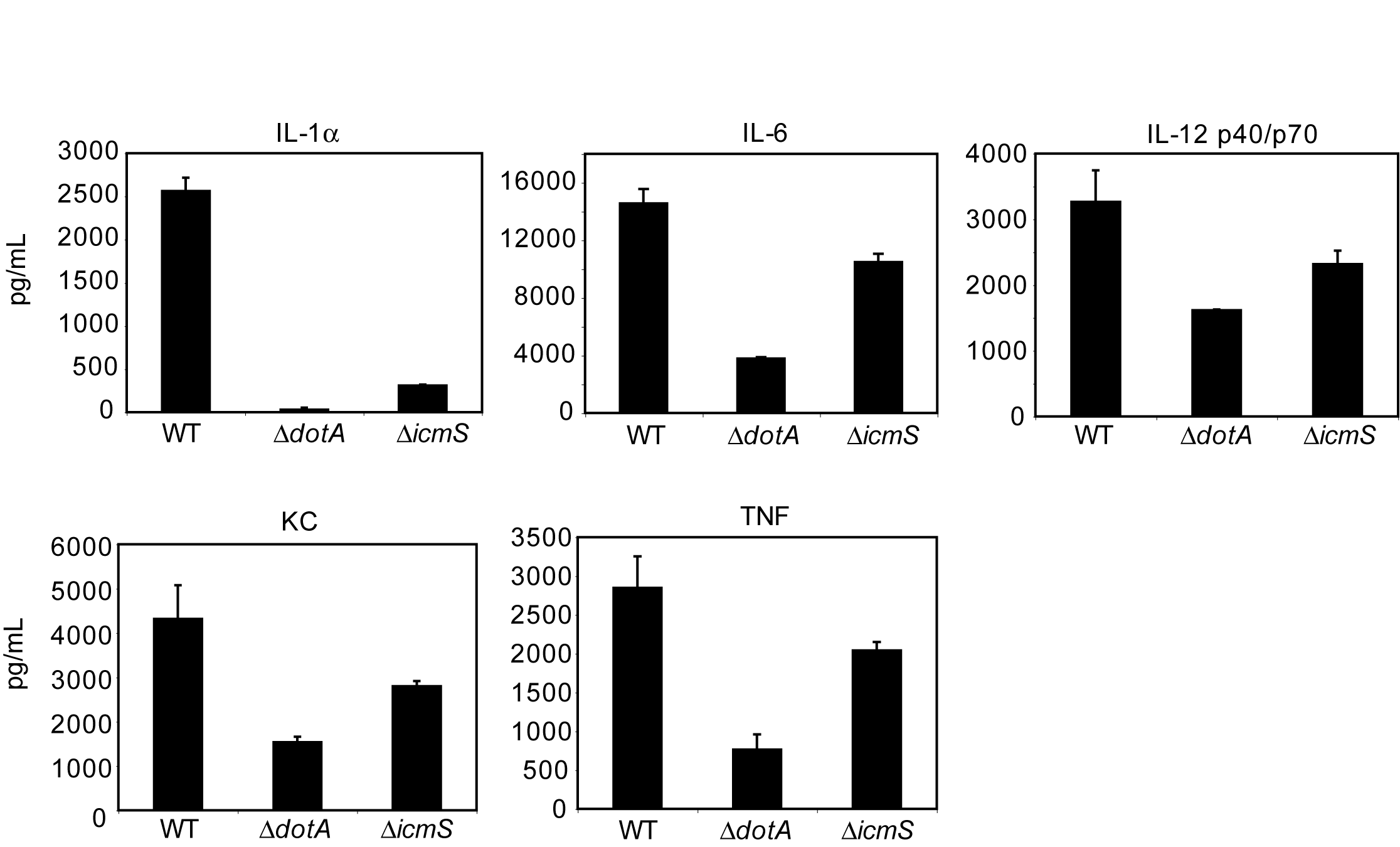

Supplement: Figure S3 — The L. pneumophila ΔicmS mutant induces slightly decreased cytokine production. (3.03 MB TIF) [file ppat.1000220.s003.tif]

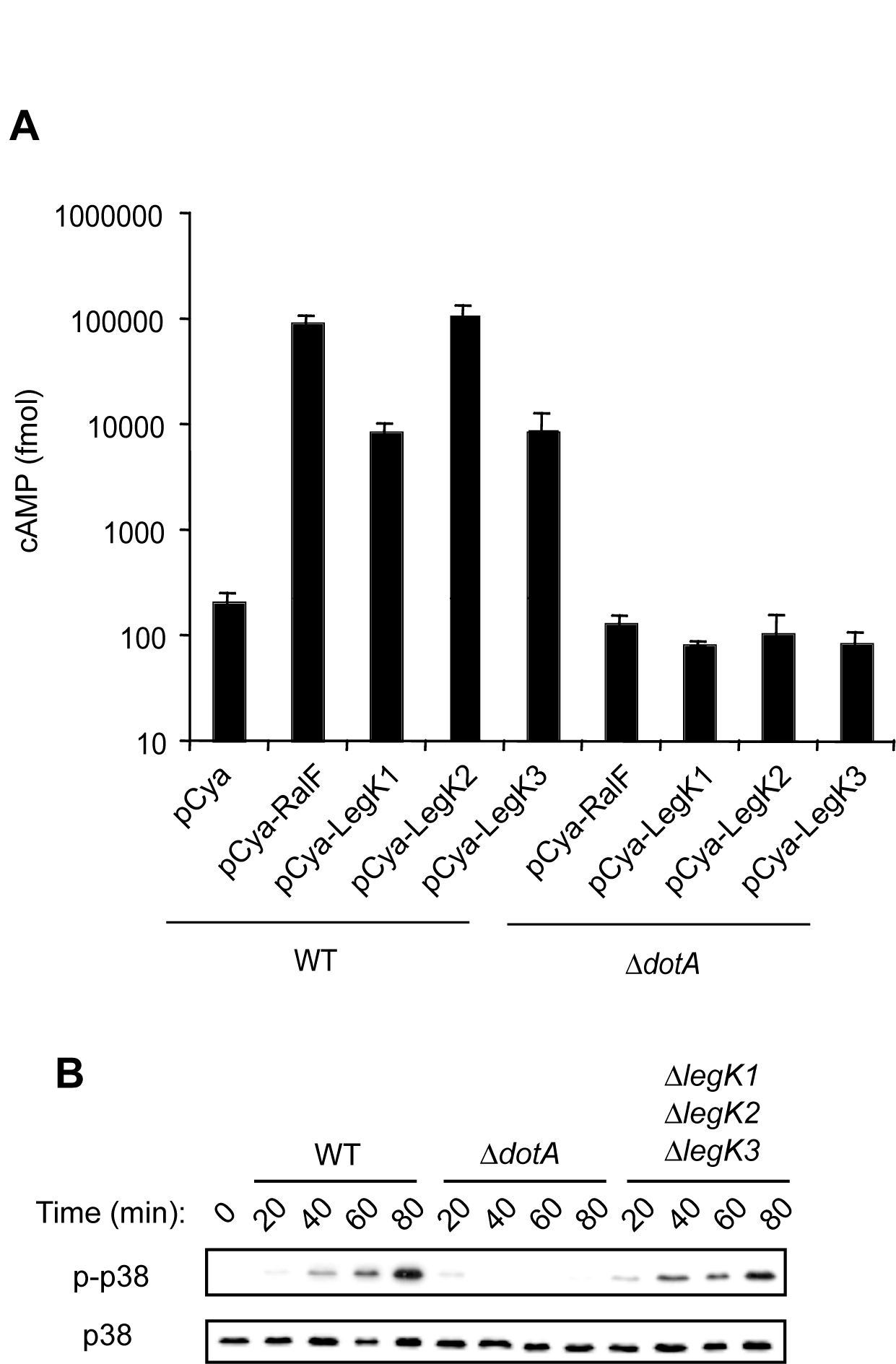

Supplement: Figure S4 — The L. pneumophila Dot/Icm system translocates three Ser/Thr protein kinases that are dispensable for p38 MAPK activation. (2.36 MB TIF) [file ppat.1000220.s004.tif]

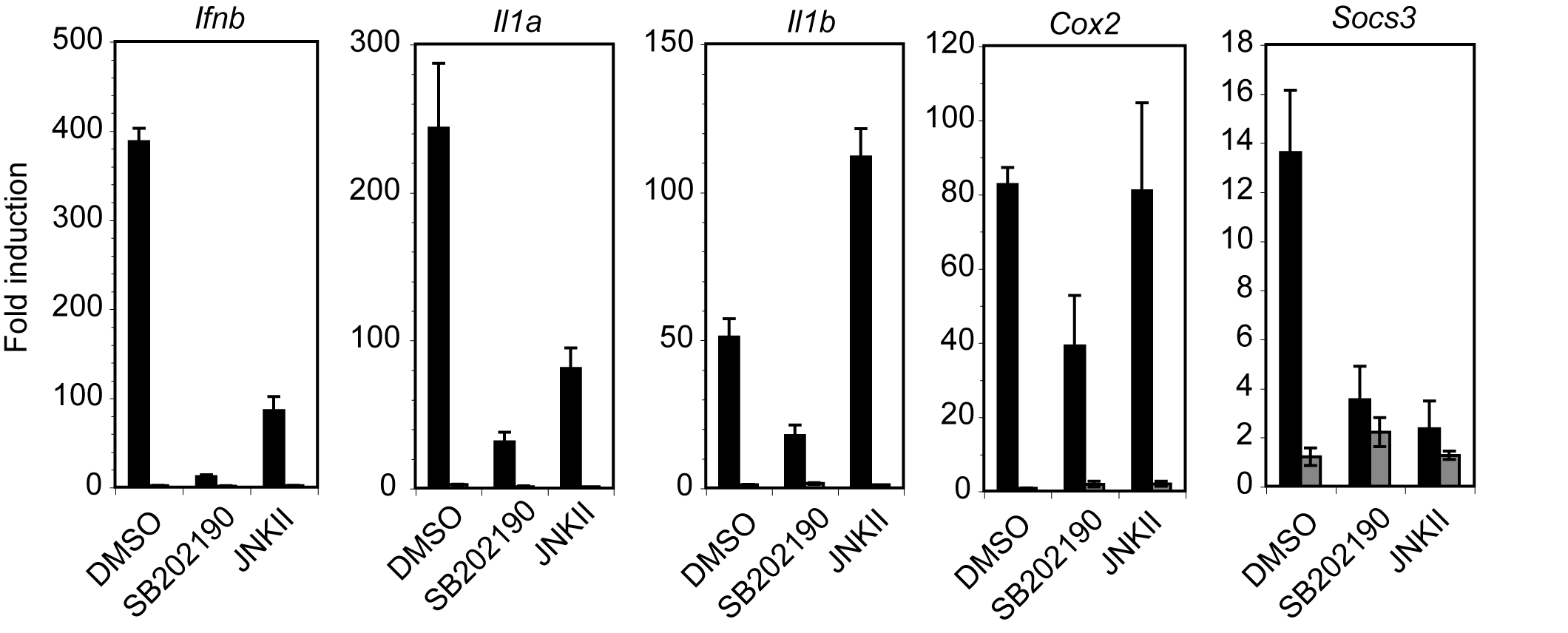

Supplement: Figure S5 — p38 and SAPK/JNK MAPK signaling contribute to Dot/Icm-dependent gene transcription in the absence of MyD88 and RIP2. (1.83 MB TIF) [file ppat.1000220.s005.tif]
